# Chemotaxis based enrichment for transgenic animals containing the *rol-6* marker

**DOI:** 10.17912/kedf-yn42

**Published:** 2018-06-07

**Authors:** John L. Carter, Rafael Morales, Steven M. Johnson, PhD

**Affiliations:** 1 Department of Microbiology and Molecular Biology, Bringham Young University, Provo, UT 84602, USA

**Figure 1.**
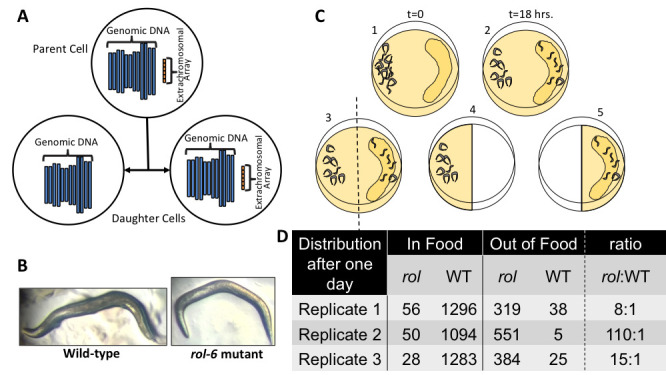
(A) Graphic depicting how *C. elegans* worms arrange transgenic elements introduced into them. (B) Pictures showing the general look of wild-type and *rol-6* mutant worms. (C) Depiction of how worms segregate via chemotaxis. (D) Data showing the number of worms, *rol-6* mutant (*rol*) vs. wild-type (WT), counted in each region, in or out of food, for three replicates, and the ratio of *rol*:WT worms in the “Out of Food” condition.

## Description

Transgenic organisms are organisms that have had foreign genes introduced into them. Making transgenic animals is a standard genetic method to test gene expression (Boulin and Hobert 2012). It is easy to make transgenic *C. elegans* by injecting a DNA construct of interest into the syncytial arm of the gonad and then looking for transgenic progeny (Evans 2006). Transgenes become arranged into extra-chromosomal arrays when introduced into the worms, and not every cell receives or propagates the array resulting in mosaic animals (Yochem and Herman 2005) (Fig.1 A).

Because the worms are mosaic, and at the outset we do not know the expression of the new transgene, we need to identify transgenic animals from their wild-type siblings without relying on the transgene. This is done with the use of co-injection markers like *rol-6(su1006)* that are injected into the worm at the same time as the transgene (Peixoto, de Melo et al 1998). Both the co-injection marker and the transgene combine together to form an extra-chromosomal array. The *rol-6* marker allows identification of transgenic progeny because the worms with the combined transgene array will roll when they move rather than moving in a sinusoidal manner like a snake (Fig.1 B). Thus, the rolling transgenic worms (“rollers”) can be individually picked and analyzed. This method of isolation is laborious and does not yield high numbers or concentrations of transgenic worms. Another method to isolate transgenic worms is to use a worm sorter that will sort worms based on fluoresces if a fluorescent marker is used. However, these machines are costly and are thus not practical for many laboratories.

*C. elegans* use chemotaxis to locate their food source. Laboratory-grown worms are fed *E. coli* strain OP50 (Brenner 1974). A large portion of the worm’s nervous system and up to 5% of the worm’s genes are dedicated to understanding and responding to environmental chemical signals (Bargmann 2006). They have elaborate behavioral patterns associated with finding and readily moving towards food (Shtonda and Avery 2006).

**Goal**Utilizing the chemotaxis of *C. elegans*, we can separate wild-type *C. elegans* from “rollers.” If a mixed population of transgenic worms harboring the *rol-6* marker, and their non-transgenic siblings are plated a distance from food, “rollers” will be unable to efficiently migrate towards the food due to impaired locomotion, while non-transgenic *C. elegans* will have normal locomotion and be able to quickly migrate to the food source. This will efficiently separate the two types of worms and will allow for the isolation of a mostly transgenic population.

**Experimental Design and Results**We set up a large 100 mm nematode growth media (NGM) plate and seeded it with *E. coli* OP50 towards the edge of the plate on just one side (Fig.1 C). We then picked and plated 15 gravid adult transgenic worms onto a single small 60 mm NGM plate previously seeded with OP50 and allowed them to grow and propagate until the plate was freshly starved. This transgenic line of *C. elegans* harbors the *rol-6(su1006)* gene in its extrachromosomal array and throws ~27% transgenic-roller worms and ~73% non-transgenic siblings. The starved plate had a mixed-stage population of offspring, and all the worms were washed off the plate using M9 buffer and then plated on the side of the 100 mm asymmetrically-seeded chemotaxis plate opposite the food. We let the worms move around for 18 hours and then observed their positions. We cut the agar from the plate in half, designated the half without food as “Out of Food” (Fig.1 C 4) and the half with food as “In Food” (Fig.1 C 5), and washed each half with M9 buffer to recover the worms. We plated the recovered worms onto two separate small seeded NGM plates. We let the worms disperse for four hours and then counted roller and non-roller worms on each plate. This experiment was replicated a total of three times on separate days. All experiments were performed at room temperature.

The non-transgenic worms migrated towards the food as expected. The rolling worms remained mostly localized to their original “Out of Food” positions (Fig.1 C). As a result, the population of worms from the “Out of Food” half was enriched for transgenic worms with at least an eight-fold *rol* to WT worm ratio in each of the three replicates (Fig.1 D). After several additional tests to determine the ideal timing, we conclude that 8-12 hours after plating on the chemotaxis plates is the best time to wash and recover the transgenic worms. This amount of time allows the worms to segregate but is not enough time for the “Out of Food” worms to starve or burrow into the agar. Additional experiments revealed that after only five hours of separation on chemotaxis plates, a significate number of the *C. elegans* found outside of the food were non-rollers (~1/3rd) resulting in only a 2:1 *rol*to WT ratio (two replicates, data not shown). Thus, five hours is not enough time to enrich for a high proportion of transgenic worms.

We were able to scale up this method to recover thousands of transgenic animals. We did this with a series of serial enrichments in which we plated worms on the chemotaxis plates, waited 8-12 hours, split the plates, washed the “Out of Food” worms off, and then fed the worms for one generation; then we repeated the same procedure again (plate, wait, split, wash, feed) with the enriched population seeded on multiple chemotaxis plates to obtain a much larger population. By doing this serial enrichment multiple times we see a tremendous increase in concentration of transgenic worms compared to the expected concentration from multiple generations of propagation without enrichment, and we are ultimately able to harvest thousands of transgenic worms with this method.

**Conclusion** We have shown that this method of enriching transgenic “rollers” is more efficient than the standard method of picking and transferring individual worms. Thus, this method saves time and facilitates the isolation of transgenic organisms for further study and applications. By using shorter chemotaxis times (8-12 hours) and feeding the worms after recovery, concerns of the worms undergoing starvation during the enrichment can be greatly allayed. Using serial enrichment, huge mixed populations that have the original proportion of transgenic worms can be obtained and then directly used or, if preferred, easily selectively picked (every fifth worm being transgenic with our line) to result in large pure transgenic populations. Additionally, this method of serial chemotactic enrichment over multiple generations could be used to screen for integrants after gamma ray-induced transgene array integration or other integration methods. We have found this enrichment method particularly useful for the biochemical and molecular analyses that we perform which require hundreds of thousands of transgenic animals.

## Reagents

NGM (nematode growth media) agar plates. OP50 *E. coli*M9 solution Any transgenic strain of *C. elegans* that contains *rol-6(su1006)* in its extrachromosomal array
